# Global Initiative for Asthma Updates for Diagnosing Asthma in Adults

**DOI:** 10.1001/jamanetworkopen.2026.11907

**Published:** 2026-05-22

**Authors:** Andrew J. Simpson, Laura Healy, Ran Wang, Miriam Bennett, Sarah Drake, Hannah Wardman, Stephen J. Fowler, Clare S. Murray, Angela Simpson

**Affiliations:** 1 School of Sport, Exercise and Rehabilitation Sciences, University of Hull, Hull, United Kingdom; 2 Division of Immunology, Immunity to Infection and Respiratory Medicine at the University of Manchester, School of Biological Sciences, The University of Manchester, Manchester, United Kingdom; 3 NIHR Manchester Biomedical Research Centre, Manchester University NHS Foundation Trust, Manchester, United Kingdom

## Abstract

This diagnostic study examines the criteria changes to the Global Initiative for Asthma (GINA) updates and their sensitivity and specificity for diagnosing asthma in adults.

## Introduction

The Global Initiative for Asthma (GINA) develops and disseminates guidance for asthma care worldwide. We previously reported that the diagnostic pathway in the GINA 2023 strategic report was associated with perfect specificity (100%) but poor sensitivity (47%).^1^ The diagnostic criterion responsible for the majority of the missed cases was airflow limitation (AFL), which was mandated in addition to evidence of expiratory airflow variability.^2^ The GINA report in 2024^3^ removed AFL as a diagnostic criterion and, in 2025,^4^ incorporated type 2 inflammatory biomarkers, to be considered when other tests are either negative or unavailable. We examine the criteria changes to GINA updates and their sensitivity and specificity for diagnosing asthma in adults.

## Methods

In this diagnostic study, symptomatic adults with general practitioner–suspected asthma, who were not treated with inhaled corticosteroids, underwent physical examination, spirometry with bronchodilator reversibility, fractional exhaled nitric oxide, peak expiratory flow variability, bronchial challenge testing, allergy testing, and blood eosinophil count testing and had treatment response assessed after 4 to 8 weeks of inhaled corticosteroids (fluticasone propionate, 250 μg twice a day), detailed elsewhere.^1,5^ The reference standard was established by a panel of asthma specialists based on this comprehensive assessment. We assessed the outcome (asthma or not asthma) for each participant against GINA 2023, 2024, and 2025 diagnostic criteria (eTable in Supplement 1) and compared them with the reference standard. The protocol was approved by the Greater Manchester East Research ethics committee; participants gave written informed consent. This diagnostic study followed the STARD reporting guideline. Analysis was performed using IBM-SPSS Statistics version 31. Data were analyzed from May 2025, to November, 2025.

## Results

In total, 118 adults (75 female; mean [SD] age, 36 [12] years) with a definitive diagnostic outcome (70 [59%] with asthma) were included between May 2019 and June 2022. Regarding the 2023 vs 2024 GINA updates, the removal of AFL increased the number of asthma diagnoses from 32 (27%) to 74 (63%) (Figure). Regarding the 2024 vs 2025 updates, the sensitivity and specificity of fractional exhaled nitric oxide level greater than 50 parts per billion and/or blood eosinophil level higher than 0.5 µL (to convert to ×10^9^/L, multiply by 0.001) for diagnosing asthma was 57% (95% CI, 45%-69%) and 88% (95% CI, 75%-95%), respectively. The addition of these type 2 biomarkers was associated with an additional 9 asthma diagnoses (8%), 5 (56%) of which were false positive. Consequently, sensitivity improved, but specificity was reduced. Overall, changes in the GINA guidelines between 2023 and 2025 increased specificity from 46% (95% CI, 34%-58%) to 96% (95% CI, 88%-99%), while sensitivity decreased from 100% (95% CI, 93%-100%) to 67% (95% CI, 52%-80%) (Table).

**Figure.**
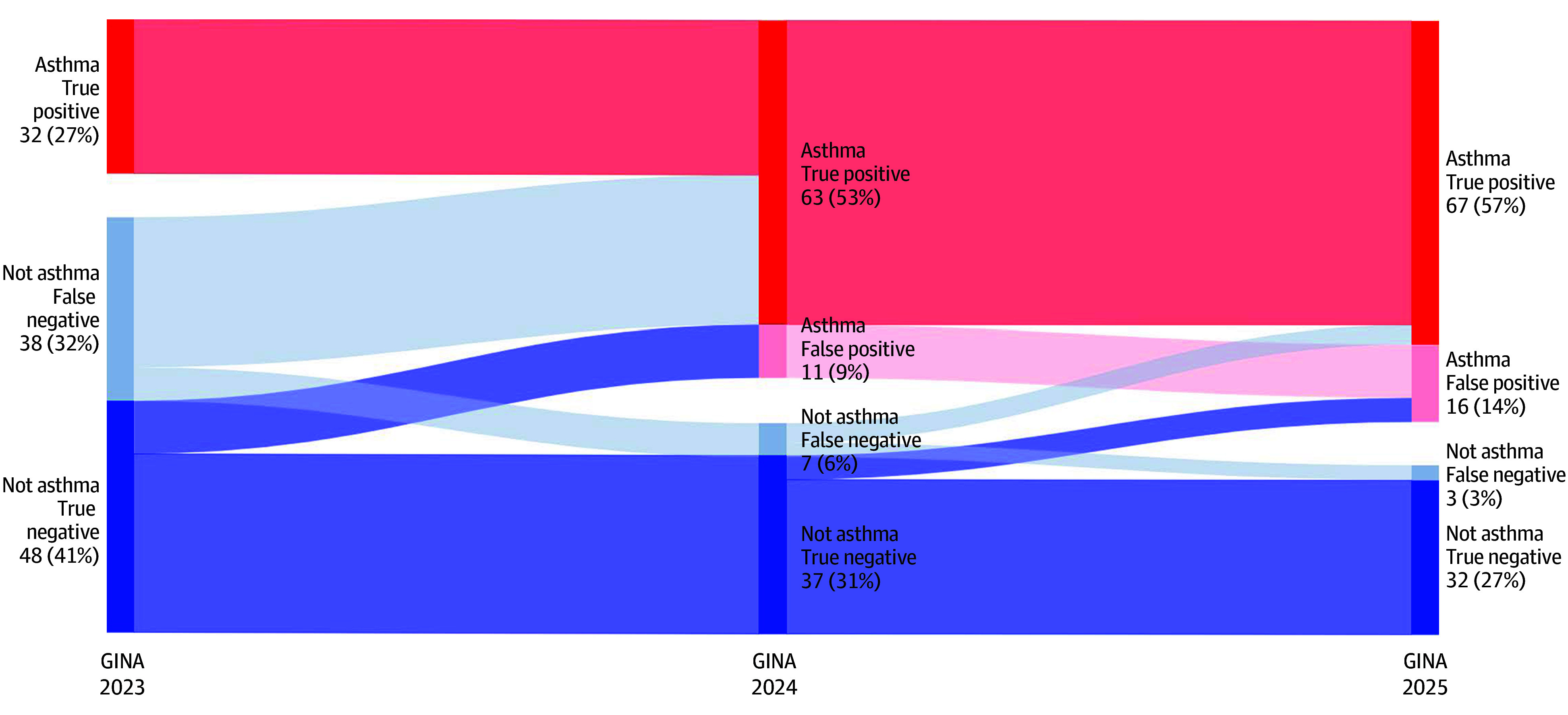
Sankey Diagram Showing the Global Initiative for Asthma (GINA) Diagnostic Criteria Changes Between 2023 and 2025 and the Asthma Diagnosis Outcomes Individual with asthma shown in red; individuals without asthma shown in blue. For each group, the number and percentage of the total for that year are given.

**Table.** Performance of 2023, 2024, and 2025 GINA Strategy Report Criteria for the Diagnosis of Asthma Compared With Expert Panel Objective Evidence Review

GINA update	% (95% CI)				
	Sensitivity	Specificity	PPV	NPV	Accuracy
2023	46 (34-58)	100 (93-100)	100 (89-100)	56 (50-61)	68 (59-76)
2024	90 (80-96)	77 (63-88)	85 (77-91)	84 (72-92)	85 (77-91)
2025	96 (88-99)	67 (52-80)	81 (74-86)	91 (78-97)	84 (76-90)

Abbreviations: GINA, Global Initiative for Asthma; NPV, negative predictive value; PPV, positive predictive value.

## Discussion

The removal of AFL (2024) and the addition of type 2 biomarkers (2025) were associated with an increase in sensitivity of GINA diagnostic guidelines for asthma (from 46% to 96%) with a concomitant reduction in specificity (from 100% to 67%). While these changes have minimized the risk of missing an asthma diagnosis, it significantly increases the likelihood of overdiagnosis. Our previous analysis demonstrated that AFL was not present in over half of asthma cases.^1^ It is unsurprising then that removing AFL as a requirement in GINA 2024 increased the sensitivity of the guideline.

Fractional exhaled nitric oxide and blood eosinophil counts are established biomarkers for type 2 inflammation. The relatively high specificity for these tests renders them a logical inclusion in the latest GINA guidelines. Introducing these biomarkers earlier in the diagnostic pathway may be more cost-effective and may reduce the need for less accessible tests.

In a diagnostic algorithm where only 1 positive result is needed to confirm asthma, overall accuracy is constrained by the test with the lowest specificity. Previous analyses report that the GINA diagnostic criterion of peak expiratory flow variability greater than 10% has a specificity of only 76%.^1^ A limitation of this study is that there is no widely accepted criterion standard for asthma diagnosis. A detailed discussion of the reference standard applied in this study is presented elsewhere.^1^

Sequentially removing the requirement for AFL and including type 2 biomarkers has switched the GINA pathway from a “rule-in” to “rule-out” approach for diagnosing asthma. GINA 2025 guidance will rarely miss a diagnosis of asthma, but 1 in 3 of those diagnosed may not have the condition (similar to a previous study^6^). Understanding this change in performance is critical for the clinicians using this diagnostic tool. We propose that changes to future diagnostic guidance be tested prospectively and that data on performance be included within published guidance.

## Notes

Supplement 1. **eTable.** Criteria for asthma diagnosis from GINA 2023, 2024, 2025 strategic reports and the test criterion applied

Supplement 2.
Data Sharing Statement
