# Supplementary material for: Global Initiative for Asthma Updates for Diagnosing Asthma in Adults
Source: JAMA Netw Open. 2026 May 22;9(5):e2611907. doi: 10.1001/jamanetworkopen.2026.11907 (PMC13197862; doi:10.1001/jamanetworkopen.2026.11907)
Supplement: Supplement 1. — eTable. Criteria for asthma diagnosis from GINA 2023, 2024, 2025 strategic reports and the test criterion applied [file jamanetwopen-e2611907-s001.pdf]

Supplemental Online Content

Simpson AJ, Healy L, Wang R, et al. Global Initiative for Asthma updates for diagnosing asthma in adults. *JAMA Netw Open*. 2026;9(5):e2611907. doi:10.1001/jamanetworkopen.2026.11907

eTable. Criteria for asthma diagnosis from GINA 2023, 2024, 2025 strategic reports and the test criterion applied

This supplemental material has been provided by the authors to give readers additional information about their work.

© 2026 Simpson AJ et al. *JAMA Network Open.*

eTable. Criteria for asthma diagnosis from GINA 2023, 2024, 2025 strategic reports and the test criterion applied

GINA criteria for asthma diagnosis	2023	2024	2025	Test criterion applied
**Airflow limitation**	✓ **AND** confirmed expiratory airflow variability			FEV_1/FVC Ratio <LLN
**Expiratory airflow variability**	✓	✓	✓	One or more positive test required from those marked below
Positive bronchodilator reversibility	✓	✓	✓	FEV_1 ≥12% and 200 mL
Excessive variability in twice daily PEF	✓	✓	✓	mean >10%, (minimum of 5 days of PEF recordings required for valid test)
Increase in lung function after ICS treatment	✓	✓	✓	Increase in FEV_1 ≥12% and 200mL from baseline after ≥ 4-weeks ICS treatment
Positive bronchial provocation test (e.g., methacholine, mannitol, hypertonic saline, eucapnic voluntary hyperpnoea or exercise)	✓	✓	✓	Methacholine bronchial test PD_{20} <0.2mg AND/OR Mannitol bronchial test PD_{15} < 635mg
Excessive variation in lung function between visits	✓	✓	✓	FEV_1V ≥12% and 200 mL between any visit
**Type-2 biomarkers**			✓ (if tests above are negative or not available)	FeNO > 50ppb AND/OR Blood eosinophils > 0.5 x 10^9 x cells/L

© 2026 Simpson AJ et al. *JAMA Network Open.*

Abbreviations: FEV_1, forced expiratory volume in one second; FVC, forced vital capacity; BDR, bronchodilator reversibility; PD, provoking dose; PEFv, peak expiratory flow variability;. FeNO, fractional exhaled nitric oxide;

© 2026 Simpson AJ et al. *JAMA Network Open.*
