# Supplementary material for: Global Initiative for Asthma Updates for Diagnosing Asthma in Adults
Source: JAMA Netw Open. 2026 May 22;9(5):e2611907. doi: 10.1001/jamanetworkopen.2026.11907 (PMC13197862; doi:10.1001/jamanetworkopen.2026.11907)
Supplement: Supplement 2. — Data Sharing Statement [file jamanetwopen-e2611907-s002.pdf]

Data Sharing Statement

Simpson. Global Initiative for Asthma Updates for Diagnosing Asthma in Adults. *JAMA Netw Open*. Published May 22, 2026. doi:10.1001/jamanetworkopen.2026.11907

Data
Data available: No
